# Systematic review of the impact of nutrition claims related to fat, sugar and energy content on food choices and energy intake

**DOI:** 10.1186/s12889-019-7622-3

**Published:** 2019-10-15

**Authors:** Laura H. Oostenbach, Esther Slits, Ella Robinson, Gary Sacks

**Affiliations:** 1 Department of International Health, Faculty of Health, Medicine, and Life Sciences, Maastricht University, Duboisdomein 30, Maastricht, 6229 GT The Netherlands; 2 Global Obesity Centre, Deakin University, 221 Burwood Highway, Burwood, VIC 3125 Australia

**Keywords:** Nutrition claims, Influence, Food choices, Energy intake, Overweight, Obesity

## Abstract

**Background:**

As part of efforts to address high levels of overweight and obesity, the provision of nutrition information (e.g., through nutrition labels and nutrition claims) on food packages has increasingly become an important policy option. This study aimed to assess the influence of nutrition claims relating to fat, sugar, and energy content on product packaging on several aspects of food choices to understand how they contribute to the prevention of overweight and obesity.

**Methods:**

A systematic literature review was conducted using the online databases EBSCOhost Global Health, EBSCOhost Medline, ScienceDirect, Scopus, PsycINFO and Embase. Studies were included if they measured the influence of nutrition claims relating to fat, sugar, and energy content on outcomes related to body weight, and were published between January 2003 and April 2018.

**Results:**

Eleven studies were included in the review. Results showed that nutrition claims can influence the knowledge of consumers with respect to perceived healthfulness of products, as well as expected and experienced tastiness of food products – making food products with nutrition claims seem healthier and less tasty. Nutrition claims can make the appropriate portion size appear to be larger and lead to an underestimation of the energy content of food products. Nutrition claims can also influence food purchase intentions, moderated by the perceived healthfulness of the relevant food products and the health consciousness of individuals. Nutrition claims were also found to have an impact on food purchases, to influence ‘consumption guilt’ (i.e., feeling of guilt associated with eating), and to increase consumption, moderated by the weight status of individuals. These influences were shown to vary depending on the type of claim and food carrying the claim.

**Conclusions:**

There is evidence that, while nutrition claims may lead some consumers to improve their nutrition knowledge and select healthier options, it may also lead consumers to increase food consumption and overall energy intake. This may run counter to efforts to address overweight and obesity.

## Background

The World Health Organization (WHO) estimates that, worldwide, the prevalence of obesity has reached epidemic proportions [1]. This is concerning as obesity can have serious impacts on health [2], and contributes to large social and economic costs [3]. To counter this obesity epidemic, there is a need for greater preventive action.

There is strong evidence that the food environment in which individuals find themselves substantially influences their eating behaviours, and thereby ultimately influences diet composition and overall energy intake [4]. Creating a supportive food environment is therefore necessary to help consumers make healthier food choices and adopt healthier eating habits [5].

The extent to which the overall food environment affects eating behaviours depends, amongst other things, on the food selection environment and on how consumers interact with it [5]. The food selection environment can be defined as the environment in which the selection of food for purchase and/or consumption occurs [6]. Nutrition information is a key component of the food selection environment, with relevant information provided on food packages (including on the front and on the back of the package), in food advertisements, and on restaurant menus [7, 8]. From a public health perspective, a key goal of the provision of nutrition information in the food selection environment is to increase awareness and knowledge regarding the nutritional content of food products. This could be expected to lead to increased purchase and intake of healthier foods, and eventually contribute to improved health status [6].

Studies have shown that for the majority of consumers, easily accessible sources of information, such as nutrition labels, are their primary sources of nutrition information [9, 10]. Yet, most consumers do not use nutrition labels due to a lack of time, and to their difficulties in understanding the information [11]. However, this has been shown to vary by demographic factors. People with a higher level of education tend to have a better understanding of nutrition labels and are more likely to use nutrition information [12]. Women are more inclined to use nutrition information than men, women with children tend to pay more attention to nutrition information than women without children, but younger women without children may read nutrition information for weight control reasons and body image concerns [13]. Similarly, people with greater health and nutrition concerns are more likely to search for nutrition information on food packages [14].

Nutrition content claims (such as “low in fat”), or more simply, nutrition claims describe the relative or absolute level of a nutrient in a food product. They can be contrasted with health claims (such as “calcium helps build strong bones”) that describe properties of a food product or food component in relation to health or disease [15]. The use of nutrition and health claims varies between countries, with several jurisdictions such as Australia, New Zealand [16], the European Union (EU) [17], Canada [18], and the United States [19] regulating their use.

While there is extensive research on the influence of nutrition labels in general [20, 21] and on the influence of health and nutrition information on portion sizes consumed [22], the specific role of nutrition and health claims in the prevention of overweight and obesity has not yet been clearly delineated [12]. There is some evidence that, in the presence of a nutrition or health claim on the front of the package, consumers are generally less inclined to take heed of other nutrition information (e.g., nutrition panels or front-of-pack labels), and are more inclined to only use the claim [23]. Furthermore, while nutrition and health claims can be useful tools to inform food purchasing, previous reviews have shown that they can have a ‘health halo’ effect, making food products carrying claims seem healthier than they are [23, 24]. In addition, there is evidence that nutrition and health claims can influence consumers’ perceptions, potentially leading to overconsumption and lowering perceived energy intake [24, 25]. A recent meta-analysis examined the effect of nutrition and health claims on packaged food products, on adult food choices [26]. It found that claims can influence food choices as products carrying claims are more likely to be selected compared to identical products without a claim. It also suggested that this influence is similar for nutrition claims and health claims, but that the extent of the influence varies by type of food product [26]. Importantly, previous reviews [23, 24, 26] have covered claims related to a wide array of nutrients, such as omega-3 fatty acids, sodium, iron, calcium, vitamins, fibre, fat, and sugar. However, when considering overweight and obesity, key nutrition claims are related to fat, sugar, and energy content [1]. There is a lack of reviews that have specifically investigated the impact of nutrition claims (as distinct from health claims) relating to fat, sugar, and energy content on various aspects of food choices.

This study aimed to conduct a systematic review of the influence of nutrition claims relating to fat, sugar, and energy content with respect to their potential influence on the knowledge and intentions of individuals, food purchases, and consumption. The study thereby sought to address the research question: how do nutrition claims relating to fat, sugar, and energy content influence consumers’ food choices and energy intake?

## Methods

### Data sources and search strategy

The online databases EBSCOhost Global Health, EBSCOhost Medline, ScienceDirect, Scopus, PsycINFO and Embase were searched. The search terms ‘nutrition claim’, ‘low in fat’, ‘high in fat’, ‘low in sugar’, ‘high in sugar’, ‘low in calorie’, ‘high in calorie’, ‘high in energy’, ‘influence’, ‘food choice’, ‘energy intake’, and ‘obesity’, and equivalent terms were used. An overview of the search terms can be found in Additional file 1. The titles and abstracts of articles retrieved in the initial search were screened against the selection criteria (see below). Selected articles were then read in their entirety, and assessed for inclusion. Reference lists of included articles as well as articles citing any of the included studies were also reviewed using the selection criteria.

### Selection criteria

Table 1 presents the inclusion and exclusion criteria that were applied to select articles. The results were filtered by a 15-year publication period (January 2003 to April 2018), to capture all articles subsequent to the first major WHO report covering nutrition claims in 2003 [27]. The results were also filtered for the English language. The review focused on the potential impact of nutrition claims on consumer food choices rather than their potential impact on the development and/or reformulation of food products by manufacturers (e.g., formulation of a product to have a healthier nutrient composition in order to be eligible to make a claim). Studies investigating impact on product development and/or reformulation were therefore excluded.

**Table 1:** Inclusion and exclusion criteria

Inclusion criteria	Exclusion criteria
Publication period: January 2003–April 2018	Other types of nutrition labelling without the presence of nutrition claims e.g. BOP labelling systems (e.g. nutrition panels), FOP labelling systems (e.g. traffic light nutrition labelling, Health Star Rating system, warning labels), FOP symbols or endorsement schemes (e.g. green Swedish Keyhole symbol, Australian/New Zealand National Heart Foundation Tick)
Language: English	Health claims (e.g. “Calcium helps build strong bones”, “Diets containing an increased amount of both fruit and vegetables reduces risk of coronary heart disease”)
Food choices (e.g. purchases, consumption) relating to the influence of nutrition claims	Non-nutritional aspects of labelling (e.g. colour and size of the nutrition claim or package)
Nutrition claims on packaged food (e.g. “low in sugar” on a box of cereal, “0% fat” on a pot of yoghurt)	Menu labelling (e.g. nutrition information on restaurant menu boards)
Nutrition claims relating to fat, sugar, and energy content (e.g. “low-fat”, “reduced-fat”, “25% less sugar”, “less calories”)	Food service
Target population: 18+ y/o	Product development and/or reformulation by manufacturers (e.g. reducing sugar content of a food product)
Study designs: all	Nutrition claims on beverages
Study outcomes: taste perceptions, nutrition knowledge, purchases, consumption, body weight	

*BOP* back-of-pack, *FOP* front-of-pack, *y/o* years old

### Quality assessment

The Effective Public Health Practice Project (EPHPP)'s Quality Assessment Tool for Quantitative Studies was used to evaluate each article with respect to the following components: selection bias, study design, confounders, blinding, data collection methods, withdrawals and dropouts, intervention integrity, and analyses [28, 29]. Each study component was assessed as ‘weak’, ‘moderate’, or ‘strong’. A final rating was given for each study; a study was rated ‘high quality’ if none of the components were assessed as ‘weak’, ‘moderate quality’ if only one of the components was assessed as ‘weak’, and ‘low quality’ if two or more components were assessed as ‘weak’. Quality assessments were conducted independently by LO and ER, with discrepancies resolved by discussion. The inter-rater agreement was of 70% before discussion of discrepancies. Percent inter-rater agreement was calculated by dividing the total number of ratings by the number of ratings in agreement.

### Data extraction and synthesis

The key focus and concepts of each study were identified, with initial characterisation based on previous reviews in the area. Data on study design, participants, settings, intervention and effectiveness measures were also extracted using a standardised data extraction template following PRISMA guidelines [30]. These characteristics were then used to analyse the collected studies. The review and data extraction were conducted by one reviewer (LO), with methods verified by the second reviewer (GS).

## Results

### Study selection

**Fig. 1:**
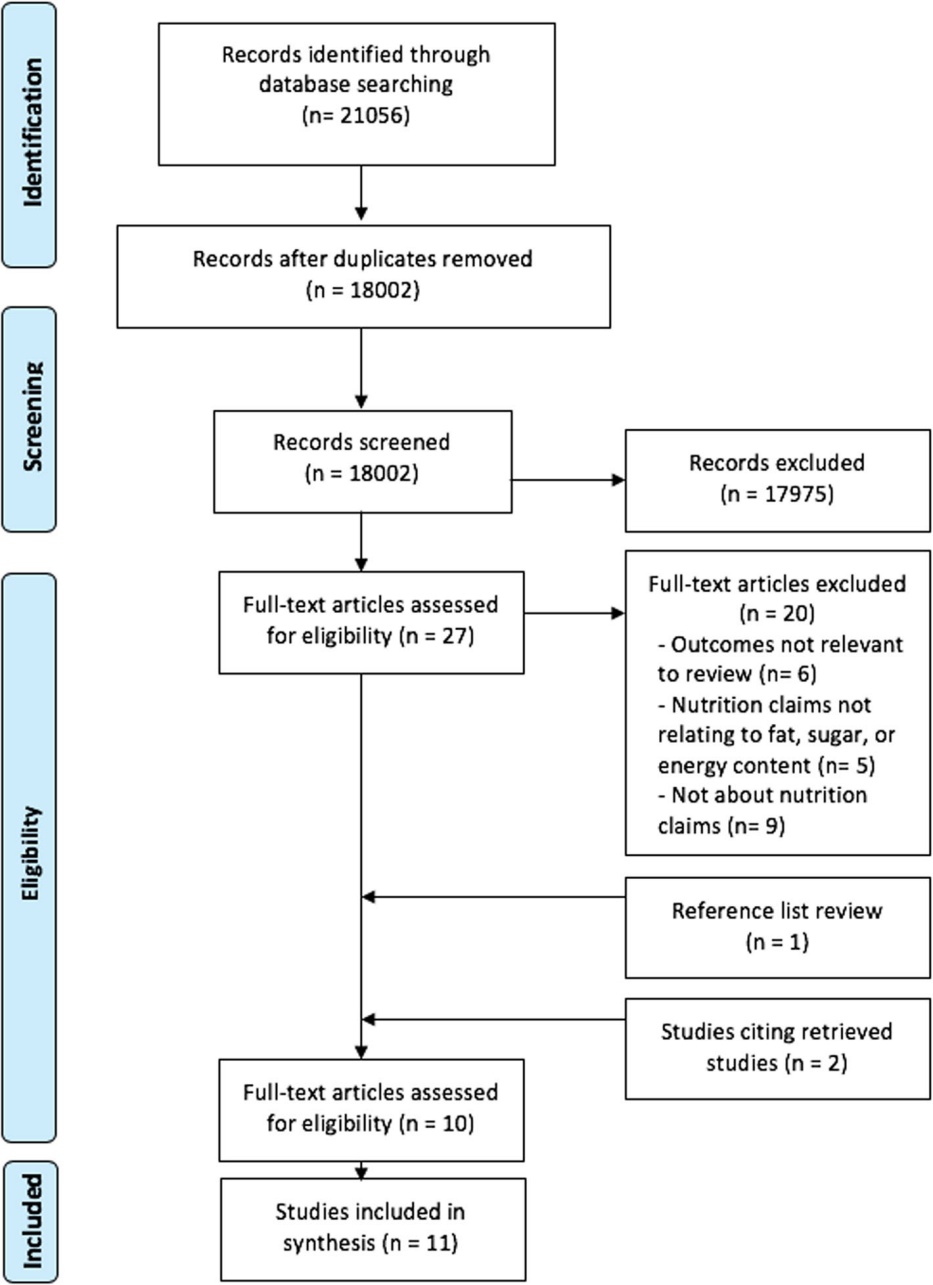
Selection process based on PRISMA guidelines [30]

The search of the literature identified 21,056 potential articles, including 3054 duplicates. From 18,002 articles selected for further scrutiny, 17,975 were deemed ineligible as a result of title and abstract screening. The full texts of the remaining 27 articles were retrieved to determine whether they were eligible. From those 27 articles, 20 were excluded based on the predetermined inclusion criteria. One additional article was identified by reviewing reference lists of included articles. Citation searching further identified two other studies. As one article presented two different studies, a total of eleven studies were included in the review. A flow chart of the selection process is depicted in Fig. 1.

### Study characteristics

Table 2 gives an overview of the country, setting, and sample size of each study. It describes which food category, and type of nutrition claim each study examined, and also indicates the outcome areas on which each study focused. Five studies were from the United States [37–40], one from Australia [31], and five from European countries, including two from Germany [32, 33], two from the United Kingdom [35, 36], and one from the Netherlands [34]. Eight studies analysed the influence of nutrition claims relating to fat [31, 34–36, 38–40], two focused on nutrition claims relating to fat and sugar [32, 33], and one on nutrition claims relating to fat as well as energy content [37]. The majority of studies were experimental. The methodological quality of most studies was ‘low’ [32–36, 38–40]. Only one study was of ‘moderate’ quality [37]. One study [31] was qualitative and was not assessed for quality because the selected tool (EPHPP) is for quantitative studies. Additional file 2 presents the key findings of each study.

**Table 2:** Overview of each study included in the review

Authors, Year	Setting	Country	Population (sample size, n)	Food category	Type of claim	Outcome areas of focus
Chan et al., 2005 [31]	Content analysis of transcript	AU	20–80 y/o (36)	• Any food	• Low-fat	• Food intake • Purchases
Bialkova et al., 2016 [32]	Experimental	DE	18–64 y/o (240)	• Chips^a^ • Cereals^a^	• 30% less fat (chips) • 30% less sugar (cereals) vs. no claim	• Experienced tastiness • Purchase intentions
Mai & Hoffmann, 2015 (study 3) [33]	Experimental	DE	av. 21.3 y/o (475)	• Yogurt^b^	• Reduced-fat • Reduced-sugar vs. Regular	• Health consciousness • Perceived healthfulness • Experienced tastiness • Purchase intentions
Roefs & Jansen 2004 [34]	Experimental	NL	women (44)	• Milkshake^a^	• Low-fat vs. High-fat	• Food intake
Faulkner et al., 2014 [35]	Experimental	UK	av. 26 y/o 21–44 y/o (186)	• Coleslaw	• Reduced-fat vs. Standard	• Perceived appropriate portion size • Perceived energy content
Norton et al., 2013 [36]	Experimental	UK	av. 24.3 y/o 18–60 y/o (87)	• Milk chocolate^a^	• Reduced-fat vs. no claim	• Perceived tastiness • Experienced tastiness
Andrews et al., 2009 [37]	Experimental	US	18+ y/o (480)	• Chocolate	• Half-the-fat • Half-the-calories vs. no claim	• Perceived healthfulness
Belei, et al., 2012 (study 1) [38]	Experimental	US	undergraduate students (109)	• Chocolate^a^	• Low-fat vs. Regular	• Food intake
Ebneter et al., 2013 [39]	Experimental	US	women av. 20.86 y/o (175)	• M&M's.^a^	• Low-fat vs. Regular	• Perceived energy content • Perceived healthfulness
Wansink & Chandon, 2006 (study 2) [40]	Experimental	US	av. 38 y/o (74)	• M&M's. • Granola bar^a^	• Low-fat vs. Regular	• Perceived appropriate portion size • Perceived energy content • Consumption guilt • Weight status
Wansink & Chandon, 2006 (study 1) [40]	Real-word setting	US	18+ y/o (269)	• M&M's.^a^	• Low-fat vs. Regular	• Food intake • Weight status

^a^No difference between the food products: the study used similar products with the same food composition

^b^Actual difference between the food products: the study used products with different food composition corresponding to the nutrition claim

*AU* Australia, *CA* Canada, *DE* Germany, *NL* The Netherlands, *UK* The United Kingdom, *US* The United States of America, *y/o* years old, *av*. average, vs. versus

### The influence of nutrition claims

#### Influence on perceived healthfulness of products

Three studies assessed the influence of nutrition claims on perceived healthfulness of products [33, 37, 39]. Andrews et al. focused on nutrition claims relating to fat and energy content on chocolate bars [37]. A ‘half-the-fat’ or ‘half-the-calories’ claim led 22% of participants to perceive chocolate bars as healthy for them, whereas when no claim appeared no participants perceived chocolate bars to be healthy [37]. Similar results were found for M&M's. (brand of chocolates) labelled as ‘low-fat’ among women [39]. Women perceived M&M's. labelled as ‘low-fat’ to be healthier than M&M's. labelled as ‘regular-fat’ [39]. Mai and Hoffmann indicated that health consciousness moderated how ‘reduced-fat’ and ‘reduced-sugar’ claims influenced healthfulness perceptions [33]. Health-conscious participants perceived ‘regular’ yogurt as less healthy than yogurt labelled as ‘reduced-fat’ or ‘reduced-sugar’. In contrast, less health-conscious participants did not perceive ‘regular’ yogurt as less healthy than ‘reduced-fat’ or ‘reduced-sugar’ yogurt [33].

#### Influence on expected and experienced tastiness

Two studies assessed the influence of nutrition claim on tastiness [32, 36]. Norton et al. focused on the expected as well as experienced tastiness of milk chocolate based on a nutrition claim relating to fat [36]. They observed that a ‘reduced-fat’ claim led consumers to believe and expect milk chocolate to not be as tasty as the ‘regular’ milk chocolate [36]. Yet, no difference in experienced tastiness between milk chocolate labelled ‘reduced-fat’ or ‘regular’ was found [36]. Similar results regarding no difference in experienced tastiness in relation to a nutrition claim were found for cereals labelled as ‘30% less sugar’ compared to cereals without a claim [32]. However, Bialkova et al. found that chips labelled as ‘30% less fat’ were experienced as less tasty than chips without a claim, even though the chips were identical [32].

#### Influence on perceived appropriate portion size and calorie estimation

Two studies focused on perceived appropriate portion size as well as perceived energy content [35, 40]. One study focused only on perceived energy (calorie) content [39]. Ebneter et al. observed that M&M's. labelled ‘low-fat’ were believed to contain 50 cal less than when a claim did not appear [39] Wansink and Chandon found that participants exposed to a ‘low-fat’ claim expected M&M's. and granola bars to contain fewer calories as compared to participants exposed to a ‘regular’ claim [40]. As a result of these underestimations, participants exposed to a ‘low-fat’ claim believed that the appropriate portion size was 25% larger as compared to participants exposed to a ‘regular’ claim [40]. Similar results regarding calorie underestimation were observed by Faulkner et al. for a ‘reduced-fat’ claim on coleslaw [35]. Consumers underestimated calorie content by 49% compared to the actual calorie content of the coleslaw with the ‘reduced-fat’ claim. The ‘reduced-fat’ claim further influenced the perception of appropriate portion size. The appropriate portion size was estimated to be larger for coleslaw labelled as ‘reduced-fat’ than for coleslaw labelled as ‘standard’. Moreover, the claim ‘reduced-fat’ led participants to believe that the appropriate portion size of coleslaw was 71% larger than the recommended portion size [35].

#### Influence on purchases and purchase intentions

Three studies examined the influence of nutrition claims on purchasing [31–33]. Chan et al. explored consumers’ beliefs and attitudes to nutrition claims relating to fat on food products [31]. Consumers reported being influenced by ‘low-fat’ claims in their purchases as they would generally want to try food products labelled “low-fat” [31]. Furthermore, Bialkova et al. assessed the influence of a ‘30% less fat’ claim and ‘30% less sugar’ claim on the stated intention to purchase chips and cereals, respectively [32]. Participants indicated a lowered intention to buy ‘30% less fat’ chips than chips without a claim, however a ‘30% less sugar’ claim on cereals did not alter participants’ stated intention to buy those cereals [32]. Mai and Hoffmann noted that a ‘reduced-fat’ claim on yogurt influenced participants’ purchase intentions through perceived healthfulness [33]. When ‘reduced-fat’ yogurt was perceived as healthy, purchase intentions increased. Higher levels of health consciousness further magnified this positive influence on purchase intentions [33].

#### Influence on food consumption and calorie intake

Five studies focused on the influence of fat-related claims on food consumption [31, 34, 38, 40]. Only one of them investigated the influence of fat-related claims on energy (calorie) intake [40]. In the study by Chan et al., participants reported viewing ‘low-fat’ claims as ‘permission’ to eat more [31]. Participants also stated that ‘low-fat’ claims caused them to eat more ‘low-fat’ foods than similar ‘regular’ foods [31]. Roefs and Jansen assessed the influence of a ‘low-fat’ versus a ‘high-fat’ claim on the intention to consume (identical) milkshakes [34]. After tasting the milkshakes, on average, all participants reported higher intentions to consume the milkshake labelled ‘low-fat’ compared to the milkshake labelled ‘high-fat’ [34]. Furthermore, Belei et al. pointed out that a ‘low-fat’ claim can increase consumption of a chocolate bar [38]. Participants in the ‘low-fat’ claim condition consumed on average 8 g more chocolate than participants in the ‘regular’ claim condition [38]. Correspondingly, Wansink and Chandon observed that during an open-house reception participants ate 28% more M&M's. (representing an additional 54 cal) when they were labelled ‘low-fat’ compared to when they were labelled as ‘regular’ [40]. Wansink and Chandon also found that a ‘low-fat’ claim led to greater M&M's. intake among participants with overweight as compared to those with normal weight [40]. Additionally, Wansink and Chandon highlighted that ‘low-fat’ claims on M&M's. and granola bars can reduce ‘consumption guilt’ associated with eating those foods [40]. ‘Low-fat’ claims lowered guilt associated with eating a granola bar among participants with normal weight as well as among participants with overweight, whereas ‘low-fat’ claims only reduced guilt associated with eating M&M's. among participants with overweight [40]. None of the studies measured the impact on consumption beyond the immediate choice at hand. None looked at how daily energy intake was affected or assessed whether compensatory behaviours occurred. No study looked at the influence of nutrition claims on weight-related outcomes (e.g., body weight, body mass index, weight status).

## Discussion

This review provided a comprehensive overview of the evidence regarding the influence of nutrition claims relating to fat, sugar, and energy content on food choices and energy intake. Results showed that nutrition claims relating to fat, sugar, or energy content can shape the knowledge of consumers with respect to perceived healthfulness of products, as well as expected and experienced tastiness of food products – making food products with nutrition claims generally seem healthier and less tasty. Nutrition claims can also make the appropriate portion size appear to be larger and lead to an underestimation of the energy content of food products. Nutrition claims can influence food purchase intentions, moderated by the perceived healthfulness of the relevant food products and the health consciousness of individuals. Nutrition claims were also found to have an influence on food purchases and on ‘consumption guilt’ associated with eating a food product, and to increase consumption, moderated by the weight status of individuals. These influences were shown to vary depending on the type of claim and food carrying the claim.

These results align with the findings of previous reviews that have shown that nutrition and health claims can have ‘health halo’ effects where consumers perceive products carrying such claims as lower in calories and healthier than they are [23, 24]. Williams found that ‘health halos’ may discourage consumers from reading more comprehensive nutrition information on labels [23]. Chandon concluded that nutrition and health claims can influence consumers’ perceptions, increasing consumption and lowering perceived energy intake [24]. Further, a recent meta-analysis on the influence of nutrition and health claims on food choices showed that foods carrying claims are 75% more likely to be chosen than identical products without a claim [26]. However, the meta-analysis showed that nutrition and health claims had a larger influence on food products categorised as ‘beans, pulse, fish, eggs, meat and other proteins’ or ‘fruits and vegetables’ as compared to ‘foods high in fat and/or sugar’ or other categories of food products [26].

**Fig. 2:**
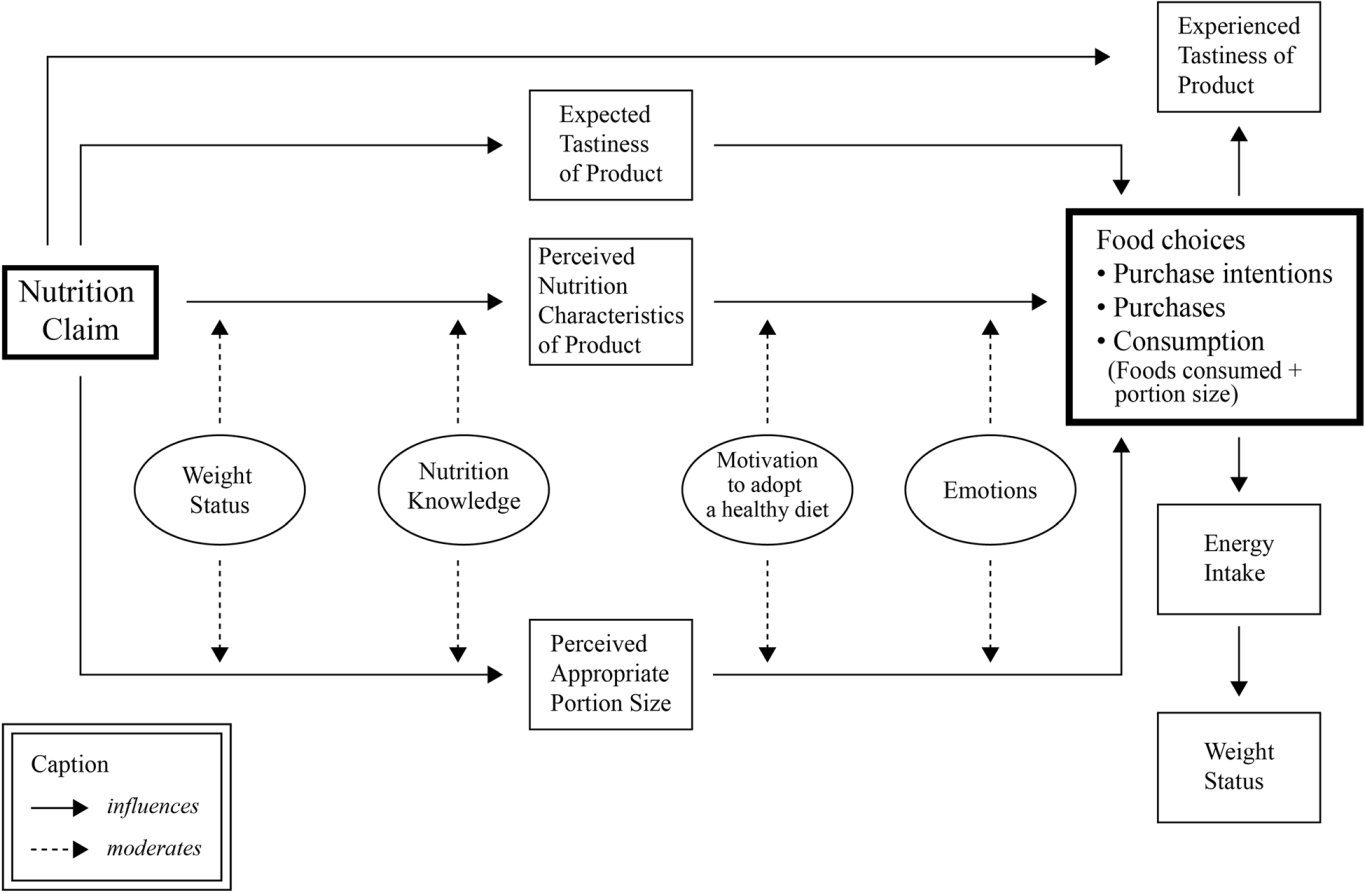
Proposed conceptual model of the potential influence of nutrition claims on food choices

The results of the review have been summarised into a conceptual model regarding the potential influence of nutrition claims on food choices. The model is presented in Fig. 2.

As the proposed conceptual model (Fig. 2) suggests, nutrition claims may influence expected and experienced tastiness of food products, perceived nutrition characteristics of food products as well as perceived appropriate portion size of food products [32, 33, 35–37, 39, 40]. Perceived nutrition characteristics of food products include perceived healthfulness of food products [33, 37, 39]. Both weight status and nutrition knowledge may moderate how nutrition claims influence nutrition characteristics perceptions of food products and the perceptions of appropriate portion size [41]. Subsequently, expected tastiness, perceived nutrition characteristics, and perceived appropriate portion size may potentially influence food choices [31, 34, 35, 38–40]. Motivation to adopt a healthy diet as well as emotions might further moderate how both nutrition characteristics perceptions of food products and the perceptions of appropriate portion size may influence food choices [33, 41]. Previous studies on cognitive and emotional influences have also shown that feelings can affect decision-making [42–44]. Guilt was found to guide and influence decisions [45, 46]. Burnett and Lunsford pointed out that health guilt or the absence of it can influence purchase as well as consumption decisions [45]. They explained that health guilt occurs when consumers believe that their decisions are not beneficial to their health [45]. Besides, other emotions such as sadness and happiness, can affect decision-making [44]. Comparably, the conceptual model suggests that food choices may vary depending on the moderating influence of emotions such as ‘consumption guilt’. Food choices can encompass purchase intentions, purchases, and consumption [31–34, 38, 40]. Consumption is understood as the foods consumed and the portion size of the foods consumed [31, 34, 38, 40]*.* Food choices (type and portion size of foods consumed) may further influence experienced tastiness and energy intake. Energy intake, in turn, predicts weight status [47, 48]. This model should be interpreted cautiously. While the proposed model suggests the potential influence of nutrition claims on food choices, the strength of each influence requires confirmation and quantification through further research.

This is the first systematic review focusing on specific nutrition claims. Methodological strengths of this review were its systematic nature and the use of the EPHPP tool to assess the methodological quality of the studies [28, 29]. However, there were several limitations with the nature of the evidence included in this review. Firstly, the methodological quality of most studies included in the review was low. This was mostly due to potential selection bias and relevant information (for quality assessment) not being reported. Information on validity and reliability of data collection tools was missing in many studies [34–36, 38–40]. Five studies had a relatively small sample size (*n* < 150) which may limit the generalizability of their findings [31, 34, 36, 38, 40]. Secondly, all studies besides one [40] were conducted in a laboratory setting that may not represent how consumers respond in ‘real-world’ situations [49]. Thirdly, all of the included studies focused on nutrition claims relating to fat, with only a small number also looking at nutrition claims relating to sugar (*n* = 2) and energy content (*n* = 1). This may limit the generalizability of the results relating to the influence of nutrition claims (not related to fat) on food choices. Generalizability of the results may be further limited because the majority of food categories assessed in the included studies were snack foods, i.e. chocolate, chips, cookies, M&M's., granola bars, and milkshakes. Only three studies focused on ‘healthier’ food products such as yogurt, coleslaw, and cereals [32, 33, 35]. More studies are needed in different food categories to draw general conclusions about the influence of nutrition claims. A fourth limitation is that only a few studies measured energy intake and studies typically only looked at the impact on selected aspects of diet. For example, several studies looked at single purchase or consumption decisions but did not assess energy in a meal, overall daily energy intake or diet quality. Thus, the overall impact of nutrition claims on daily energy intake is largely unknown. Further research needs to look at the impact on daily intake and identify potential compensatory behaviours.

There were also a number of limitations associated with the review methods themselves. The review only included articles written in English, and therefore relevant studies in other languages may have been excluded. Furthermore, as the review focused on the isolated influence of nutrition claims, conclusions regarding the influence of nutrition claims should be drawn cautiously as many other factors such as availability, affordability, and cultural differences may influence food consumption [50–52]. In addition, this review did not examine the impact on consumers’ beverages choices as it focused on nutrition claims on food products. Further, this review did not look at the potential impact on supply-side factors such as product development and reformulation. The way nutrition claims are displayed (e.g. size and colour) and their interaction with other components of the package (e.g. front-of-pack (FOP) labelling systems, warning labels) needs to be investigated. Moreover, the way in which nutrition claims interact with other factors within the food environment such as food advertising and food formulation need to be considered and further investigated to better understand how the food environment influences overweight and obesity.

### Implications for policy makers

Policy approaches contributing to the prevention of overweight and obesity through the food selection environment have focused on providing nutrition information to promote healthier eating behaviours. Results of this review indicate that nutrition claims relating to fat, sugar, and energy content are likely to increase purchase intentions when food products are perceived as healthier. However, there are indications that they may also have the unintended consequence of leading to energy overconsumption.

Although the quality of the evidence included in this review is low, and the results are indicative at best, the current evidence suggests that policy makers may need to exercise caution regarding nutrition claims relating to fat, sugar and energy content due to their potential negative influences on the healthiness of food choices and, consequently, population weight outcomes. In Australia, before any health claim can be made, the food product must meet a certain level of healthiness determined by a detailed set of nutrient profiling criteria. However, the same criteria do not apply to nutrition claims [16]. In the EU, the use of nutrition and health claims is permitted, subject to regulations regarding the specific claims that can be made [17]. The regulations planned to incorporate specific nutrient profiles for the use of nutrition and health claims. The regulations planned for the level of certain nutrients contained in a food product as well as the role and importance of that product in a healthy diet to be eligibility criteria for permitting nutrition and health claims. However, the criteria have not yet been proposed and have thus not yet been applied [17, 53]. Given the potential negative influence of nutrition claims and that such claims are in principle regulated to prevent any practices that may mislead consumers in their food purchases, governments could consider options to limit potential negative influences of nutrition claims, such as by preventing their use or only allowing their use on food products that meet specific measures of healthiness. Moreover, if nutrition claims are used, policy makers could consider making it mandatory to have interpretive FOP labels (e.g., Health Star Rating or warning labels) that can give an overall impression of the product’s healthfulness. This will help to ensure consumers have, for every food product, concise and useful nutrition information at their disposal to make healthier food choices [8, 15, 54].

A recent meta-analysis assessing the impact of nutrition labelling on food choices showed that nutrition labelling may be an effective approach in steering consumers’ food choices towards healthier products [20]. Although nutrition claims are one aspect of nutrition labelling, policy makers need to consider all aspects of labelling, including FOP symbols and interpretive labelling, warning labels, back-of-pack nutrition information panels, and claims. A potential alternative to nutrition claims may be Chile’s warning labels that flag food products with high content of key nutrients to discourage consumption of unhealthy food products [55], although their impact needs to be evaluated. Importantly, all forms of nutrition labelling are likely to be only a minor influence on diets overall. Accordingly, policies on nutrition claims need to be only one part of a comprehensive strategy to improve population diets and address obesity [56–59].

## Conclusion

This study reviewed the evidence regarding the influence of nutrition claims relating to fat, sugar, and energy content on consumers’ food choices. Findings indicated that nutrition claims may have an impact on the knowledge of consumers with respect to perceived healthfulness, expected and experienced tastiness, and perceived appropriate portion size. Nutrition claims were found to potentially influence food purchase intentions, food purchases and consumption. The findings also indicated the potential for unintended consequences, whereby nutrition claims may lead to overconsumption of foods and subsequent higher energy intakes. Using the precautionary principle, policy makers should consider options to limit potential negative influences of nutrition claims.

## Supplementary information

**Additional file 1.** Concepts and search terms used to find studies for the review.

**Additional file 2.** Summary of the studies assessing the influence of nutrition claims relating to fat, sugar, and/or energy content.

## Data Availability

Not applicable.

## Abbreviations

EPHPP: Effective Public Health Practice Project
EU: European Union
FOP: front-of-pack
WHO: World Health Organization
