# Supplementary material for: Systematic review of the impact of nutrition claims related to fat, sugar and energy content on food choices and energy intake
Source: BMC Public Health. 2019 Oct 15;19:1296. doi: 10.1186/s12889-019-7622-3 (PMC6794740; doi:10.1186/s12889-019-7622-3)
Supplement: Supplementary file 1 — Additional file 1. Concepts and search terms used to find studies for the review. [file 12889_2019_7622_MOESM1_ESM.docx]

| **Additional file 1:** Concepts and search terms | |
| --- | --- |
| **Concepts** | **Search terms** |
| 1. Nutrition claim | ‘nutrition* claim*’ or ‘fat claim*’ or ‘sugar claim*’ or ‘energy claim*’ or ‘diet claim*’ or ‘light claim*’ |
| 2. Fat or sugar | (reduc* or low*) N2 (fat or sugar) |
| 3. Energy | energy or calori* |
| 4. Influence | influenc* or effect* or impact* or affect* |
| 5. Food choice | ‘food choice*’ or ‘food consum*’ or ‘food preference*’ or ‘food habit*’ |
| 6. Energy intake | ‘energy intake*’ or ‘energy consum*’ |
| 7. Obesity | obes* or overweight or ‘weight gain*’ or ‘weight status’ |
