# Supplementary material for: Systematic review of the impact of nutrition claims related to fat, sugar and energy content on food choices and energy intake
Source: BMC Public Health. 2019 Oct 15;19:1296. doi: 10.1186/s12889-019-7622-3 (PMC6794740; doi:10.1186/s12889-019-7622-3)
Supplement: Supplementary file 2 — Additional file 2. Summary of the studies assessing the influence of nutrition claims relating to fat, sugar, and/or energy content. [file 12889_2019_7622_MOESM2_ESM.docx]

| **Additional file 2:** Summary of the studies assessing the influence of nutrition claims relating to fat, sugar, and/or energy content | | | | | | | | |
| --- | --- | --- | --- | --- | --- | --- | --- | --- |
| **Author** | **Setting** | **Country** | **Population**  **(sample size, n)** | **Food category** | **Type of claim** | **Focus** | **Key findings** | **Study Quality** |
| Chan et al., 2005 | Content analysis of transcript | AU | 72% female  20-80y/o (36) | - Any food | - Low-fat | - Food intake - Purchases | - ‘Low-fat’ was interpreted as a permission to eat more - Consumers believed that ‘low-fat’ can trigger them to eat more of ‘low-fat’ products than similar ‘regular’ products - Consumers reported being influenced by ‘low-fat’ claims in their purchasing and reported generally trying ‘low-fat’ products | Not  assessed |
| Bialkova et al, 2016 | Experimental | DE | 57% female  18-64y/o (240) | - Chips* - Cereals* | - 30% less fat (chips) - 30% less sugar (cereals)   vs. no claim | - Experienced tastiness - Purchase intentions | - ‘30% less fat’ chips were experienced as less tasty than when no claim appeared - ’30% less sugar’ on cereals made no difference in taste experience - ‘30% less fat’ lowered consumers’ intention to buy those chips - ‘30% less sugar’ did not change intentions to buy cereals | Weak |
| Mai & Hoffmann, 2015  (study 3) | Experimental | DE | 45.7% female  av. 21.3y/o (475) | - Yogurt** | - Reduced-fat (0.1%) - Reduced-sugar (-30%)   vs. Regular | - Health consciousness - Perceived healthfulness - Experienced tastiness - Purchase intentions | - Health-conscious consumers perceived ‘reduced-sugar’ or ‘reduced-fat’ products to be healthier than ‘regular’ products - ‘Reduced-fat’ influenced purchase intentions through perceived healthfulness; when ‘reduced-fat’ yogurt was perceived as healthy, purchase intentions increased - Higher levels of health consciousness enhanced the positive effect of perceived healthfulness on purchase intentions | Weak |
| Roefs & Jansen 2004 | Experimental | NL | women (44) | - Milkshake* | - Low-fat   vs. High-fat | - Food intake | - Participants reported higher intentions to consume ‘low-fat’ milkshake compared to ‘high-fat’ milkshake in the future | Weak |
| Faulkner et al., 2014 | Experimental | UK | 52% female  21-44 y/o  av. 26y/o (186) | - Coleslaw | - Reduced-fat vs. Standard | - Perceived appropriate portion size - Perceived energy content | - Perception of appropriate portion size were larger for the ‘reduced-fat’ coleslaw than that of the ‘standard’ coleslaw - Portion size was estimated to be 71% larger than the recommended serving size - ‘Reduced-fat’ coleslaw was perceived to be less caloric than ‘regular’ coleslaw - **‘**Reduced-fat’ led to an underestimation of calorie content by 49% compared to the actual calorie content | Weak |
| Norton et al, 2013 | Experimental | UK | women  18-60y/o  av. 24.3y/o (87) | - Milk chocolate* | - Reduced-fat vs. no claim | - Perceived tastiness - Experienced tastiness | - ‘Reduced-fat’ led participants to believe that the chocolate did not taste as good as ‘regular’ chocolate - No difference in experience tastiness between ‘reduced-fat’ and ‘regular’ chocolate | Weak |
| Andrews et al., 2009 | Experimental | US | 18+ y/o (480) | - Chocolate | - Half-the-fat - Half-the-calories   vs. no claim | - Perceived healthfulness | - When using either a ‘half-the-fat’ or a ‘half-the-calories’ claim, 22% of participants reported perceiving the chocolate bar as generally healthy for them, whereas 0% perceived the chocolate bar to be healthy in the control groups (no claim) | Moderate |
| Belei, et al., 2012  (study 1) | Experimental | US | undergraduate students (109) | - Chocolate* | - Low-fat   vs. Regular | - Food intake | - Group in the ‘low-fat’ condition consumed on average 8g more chocolate than the group in the regular condition | Weak |
| Ebneter et al., 2013 | Experimental | US | women  av. 20.8y/o (175) | - M&Ms * | - Low-fat   vs. Regular | - Perceived energy content - Perceived healthfulness | - ‘Low-fat’ M&Ms were perceived to contain 50 calories less than ‘regular’ M&Ms - Participants believed ‘low-fat’ M&Ms to be healthier than ‘regular’ M&Ms | Weak |
| Wansink & Chandon, 2006  (study 2) | Experimental | US | 66% female  av. 38y/o (74) | - M&Ms* - Granola bar* | - Low-fat   vs. Regular | - Perceived appropriate portion size - Perceived energy content - Consumption guilt - Weight Status | - Participants who saw a ‘low-fat’ claim on M&Ms and granola bars expected them to contain fewer calories than those who saw a ‘regular’ label - Participants who saw a ’low-fat’ claim on M&Ms and granola bars expected the appropriate portion size to be 25% larger (than those who saw the ‘regular’ claim regardless of their weight status) - ‘Low-fat’ reduced consumption guilt - ‘Low-fat’ reduced guilt associated with eating granola among everyone but with eating M&Ms only among overweight participants | Weak |
| Wansink & Chandon, 2006  (study 1) | Real-word setting (open-house reception) | US | 18+ y/o (269) | - M&Ms* | - Low-fat   vs. Regular | - Food intake - Weight status | - Participants ate 28.4% more M&Ms when labelled ‘low-fat’ than when labelled ‘regular’ - ‘Low-fat’ led to greater intake among participants with overweight participant as compared to participants with normal weight | Weak |

*No difference between the food products: the study used similar products with the same food composition.

**Actual difference between the food products: the study used products with different food composition corresponding to the nutrition claim.

AU = Australia; DE = Germany; NL = The Netherlands; UK = The United Kingdom; US = The United States of America; y/o = years old; av. = average; vs. = versus
